# Retraction: Role of cytochrome P450 in drug interactions

**DOI:** 10.1186/1743-7075-11-11

**Published:** 2014-02-14

**Authors:** Zakia Bibi

**Affiliations:** 1 Department of Chemistry, University of Karachi, Karachi 75270, Pakistan

## 

This article [1] has been retracted by the Editor because of extensive overlap with a previously published work [2]. The article was submitted by three authors, two of whom subsequently withdrew their names from the article. Despite efforts to contact the original submitting authors and to get their institution at the time of submission to initiate an investigation, we have been unable to establish how far the remaining author was aware of the submission.
